# The relationship between patients’ perception of type 2 diabetes and medication adherence: a cross-sectional study in Japan

**DOI:** 10.1186/s40780-019-0132-8

**Published:** 2019-01-22

**Authors:** Kana Hashimoto, Koki Urata, Ayano Yoshida, Reiko Horiuchi, Naoto Yamaaki, Kunimasa Yagi, Kunizo Arai

**Affiliations:** 1 Faculty of Pharmacy, Institute of Medical, Pharmaceutical, and Health Sciences, Kanazawa University, Kakuma-machi, Kanazawa, 920-1192 Japan; 2 Gran Pharma Inc., 1-5-2 Hon-machi, Kanazawa, 920-0853 Japan; 3 Department of Internal Medicine, Japan Community Healthcare Organization Kanazawa Hospital, Ha-15 Oki-machi, Kanazawa, 920-8610 Japan; 4 Department of Internal Medicine, Graduate School of Medical Science, Kanazawa University, 13-1 Takara-machi, Kanazawa, 920-8641 Japan

**Keywords:** Diabetes, Medication adherence, Illness perceptions, Community pharmacy, Hospital

## Abstract

**Background:**

The self-management of type 2 diabetes mellitus (T2DM), which involves adherence to medical instructions on diet and nutritional advice, physical activity, medication regimen, and weight and stress management, is necessary for the treatment of T2DM.

In this study, we investigated the relationship between patients’ perceptions of their disease and their adherence to their medications. And we attempted to determine whether distinct subphenotypes of behavioral change of medication adherence can be discerned based on a patients’ perceptions.

**Method:**

A cross-sectional study using a questionnaire was conducted among 157 patients with T2DM from October 2015 to September 2017. Questionnaires were administered to assess the participants’ demographic and clinical characteristics, medication adherence, diabetes knowledge, and perception of being diabetic. Principal component analysis (PCA) and cluster analyses were performed to classify medication adherence patterns in the total cohort. Multiple regression analyses were performed to identify the determinant factors of medication adherence.

**Results:**

PCA showed the interpretable medication adherence of patients with diabetes by using component 1 (“accessibility to medical treatment”) and component 2 (“status of taking medicines”). We identified four groups that show significantly different medication adherence by using cluster analysis on the basis of the two components. Multiple regression analysis showed that body mass index (BMI), family history of diabetes, one factor of patient’s perception (living an orderly life), and diabetes knowledge were found to be significant predictors of medication adherence in patients with T2DM.

**Conclusions:**

In patients with T2DM, the patient’s diabetes perception of “living an orderly life” is associated with medication adherence. A poor adherence group may be able to change their adherence to diabetes treatment by developing the perception of “living an orderly life.”

**Electronic supplementary material:**

The online version of this article (10.1186/s40780-019-0132-8) contains supplementary material, which is available to authorized users.

## Background

People with chronic conditions must be capable of self-management to protect their own health. Healthcare providers should provide support for patients facing health challenges who need assistance [1]. However, compliance with complex regimens and the self-care behavior of diabetic patients worsen over the long-term with lifestyle changes [2, 3]. This is a serious problem for both patients and healthcare providers. Therefore, medical staff should understand the factors influencing patients’ self-management behavior.

Self-management of type 2 diabetes mellitus (T2DM), which involves adherence to medical instructions on diet and nutrition, physical activity, medication regimen, and weight and stress management, is necessary for treating T2DM [4–6]. Adherence to diabetes therapy can improve patients’ blood glucose control and help them avoid long-term complications [1, 7–9]. Furthermore, many studies have shown a strong association between diabetes perception and diabetes control [10–12].

Adherence to medical instructions and glycemic control are affected by several factors such as knowledge about diabetes [13], self-efficacy [14–17], depression [18–20], medical beliefs [21, 22], medical cost, and social support. Disease perception is thought to be an important psychosocial factor that can motivate patients to self-manage their diabetes. Their perception of the illness is formed by the cause, duration, awareness of symptoms, and controllability of the disease, along with a patient-created schematic diagram of the disease. Many studies have demonstrated that the illness perception of diabetic patients influences their self-care behavior [23–25], but little quantitative research concerning a relationship between medication adherence and illness perceptions of T2DM was found.

Illness perception questionnaires for various medical conditions have been developed recently to examine patients’ perceptions about their diseases, symptoms, and causes [26]. Kamatani et al. [27] reported that T2DM patients form perceptions of diabetes in a similar manner to disease acceptance; they addressed this issue for T2DM in Japan by creating a new illness perception questionnaire (asking about the patient’s diabetic profile) for understanding the patients’ perception of their disease. They studied the relationship between blood glucose control and the T2DM patients’ perception of their disease. Although some patient and treatment characteristics are predictive of lower adherence in therapy, characteristics of patients’ perceptions associated with anti-diabetic treatments nonadherence remain unclear. The patient’s perceptions of diabetes may offer new insights into glycemic control variations in T2DM patients. Further, healthcare providers can focus on behavioral approaches to managing T2DM by understanding the patients’ illness perception.

The primary aim of this study was to investigate the relationship between patients’ perceptions of T2DM and medication adherence. Secondary aims were (1) to categorize a behavioral subphenotypes of medication adherence in T2DM using a custom medication adherence assessment tool, developed by Ueno et al. (2) to report the behavioral profile associated with the behavioral subphenotypes of medication adherence,, and (3) to investigate the relationship between behavioral subphenotypes of medication adherence in T2DM.

## Methods

A cross-sectional study using a questionnaire was conducted from October 2015 to September 2017. This study included adults who were (1) aged over 20 years, (2) diagnosed with T2DM for at least one year, and (3) outpatients of a community pharmacy or a hospital in the Ishikawa Prefecture. The patients were recruited at a community pharmacy (Aozora Pharmacy) or at the Japan Community Healthcare Organization (JCHO) Kanazawa Hospital and Kanazawa University Hospital. After obtaining informed written consent, data was collected using an interview questionnaire that had four domains, namely: (1) demographic and clinical characteristics, (2) medication adherence, (3) illness perceptions about diabetes, and (4) diabetes knowledge.

The experimental methods were approved by the Kanazawa University of Medicine Ethics Committee and the JCHO Kanazawa Hospital Ethics Committee. All work was conducted in accordance with the Declaration of Helsinki and ethical principles for clinical research. Written informed consent was obtained from all patients.

### Demographic and clinical characteristics

We collected data on patients’ age, sex, body mass index (BMI), diabetes duration, family history of diabetes, microvascular complications, important comorbidities, and treatment modalities (different types of insulin therapy). Their glycemic control levels (HbA1c), number of medications, number of doses per day, history of microvascular complications, and important comorbidities were obtained from the chart data. The microvascular complication status was defined as the presence of retinopathy, neuropathy, or nephropathy.

### Medication adherence

Ueno et al. [28] developed new medication adherence scale factors (subscale factor 1: collaboration with healthcare providers; subscale factor 2: motivation for collecting and utilizing medication-related information and utilization of information regarding medication; subscale factor 3: agreement with taking medication and its fit with their lifestyle; subscale factor 4: current state of medication use) and evaluated their reliability and validity. Medication adherence was assessed using the Ueno method. The medication adherence scale can be used with all items or each of the four subscale areas alone. The total score and the sum of each of the four subscale regions were calculated, and a higher score represents better medication implementation. The contents of each of the four subscale regions in the entire medication adherence were also evaluated. The medication adherence questionnaire about diabetes was shown in Additional file 1: Table S1.

### Illness perceptions about diabetes

The illness perception questionnaire, developed by Kamatani et al. [27], was used to collect data on the participants’ perceptions of their diabetes. The questionnaire consisted of 29 items and 7 factors (factor 1: feeling of inferiority; factor 2: living an orderly life; factor 3: feeling of restriction; factor 4: feeling miserable; factor 5: feeling of getting into trouble; factor 6: feeling of overindulgence; factor 7: feeling of importance). The questionnaire for illness perceptions about diabetes was shown in Additional file 2: Table S2.

### Diabetes knowledge

We used the revised Michigan Diabetes Knowledge Scale (DKT) [29] to measure the patients’ knowledge of diabetes treatment. The revised DKT comprises a 20-item tool with two subscales. The first 18 questions comprise the general diabetes knowledge section for patients with either type 1 diabetes or T2DM. The insulin use subscale comprises two items and is only appropriate for use with patients being treated with insulin. Thus, we used only the general knowledge subscale because this study enrolled T2DM patients regardless of the treatment regimen. The revised Michigan Diabetes Knowledge Scale (DKT) was shown in Additional file 3: Table S3.

### Statistical procedures and analyses

The significance of the demographic characteristic differences among the study patients was assessed using the Mann–Whitney U test or Fisher’s exact test between two sets of observations, and the Kruskal–Wallis test was performed for three or more variables. The statistical significance of the individual differences was evaluated using Haberman’s residual analysis and the Bonferroni method if the analysis of variance was significant. The relationships between the total medication adherence score and the patient characteristics were analyzed using Spearman’s rho statistic (ρ).

Principal component analysis (PCA) and cluster analyses were performed for the total cohort. Medical adherence patterns were extracted with PCA using the correlation matrix. To determine the number of retained components, the proportion of the variance explained by the components was used. In particular, two components were retained because they explained 85% of the medication adherence. For multiple group comparisons of patient perception and patient characteristics between medication adherence groups, we used analysis of variance. For correlations among age, BMI, family history of diabetes, two factors of illness perceptions about diabetes (factor 2: living an orderly life and factor 6: feeling of overindulgence), and diabetes knowledge, Spearman’s correlation coefficient was used. Subsequently, we conducted multiple regression analyses with medication adherence as the dependent variable.

Data from the questionnaires were analyzed using the Statistical Package for Social Sciences (SPSS version 25; IBM Japan, Tokyo, Japan), with a *p*-value < 0.05 considered statistically significant.

## Results

### Demographic/clinical characteristics and illness perceptions about diabetes and diabetes-specific knowledge

A total of 90 and 67 patients with T2DM participated in the hospital and community settings, respectively. The participants’ demographics in each setting are shown in Table 1**.** The two groups were comparable regarding diabetes duration, diabetes knowledge, and patient’s perceptions; however, the mean age and the proportion of the females in the community pharmacy patients were higher than those of the hospital patients. Moreover, more of the hospital patients received insulin treatments than the community pharmacy patients. The mean age for the total sample population was 65.8 years, and the sample population was comprised of 69.2% men. The patients’ median BMI was 24.0. The sex distribution and age of this sample population were different from those of the whole population of the hospital and community pharmacy. The median duration of diabetes in the sample population was 11.0 years. A positive family history of diabetes mellitus was reported by 55.3% of the sample population. Approximately 30% of the sample population reported having complications, including diabetic retinopathy (8.8%), diabetic nephropathy (6.3%), diabetic neuropathy (10.1%), and cardiac problems (11.3%). Insulin therapy was administered to 34% of the patients. The number of doses taken by the patients was 8 per day, and 5 medications were prescribed. The average total score of knowledge about the disease (general features of diabetes, diet, physical activity, and complications) was 9.8 (out of a maximum of 18 on the general knowledge subscale).

**Table 1:** Demographic and clinical characteristics of patients with type 2 diabetes at study entry

		Total		Hospital (*n* = 92)		Community pharmacy (*n* = 67)		*p*-value^a^
		*n*	%	*n*	%	*n*	%	
Gender	male	110	69.2	72	78.3	38	56.7	0.005*
	female	49	30.8	20	21.7	29	43.3	
Age (year)	median [range]	68 [32–88]		65[32–87]		70[42–88]		0.001*
BMI	median [range]	24.0 [15.2–34.8]		24.0[15.9–34.8]		24.0[15.2–34.0]		0.446
Diabetes Duration (year)	median [range]	11,0 [0–55]		12.0[0–55]		10.0[1–53]		0.447
HbA1c (%)	median [range]	7.0 [4.4–10.6]		7.0 [4.4–10.0]		7.1 [5.9–10.6]		0.364
Diabetes history of relatives	yes	88	55.3	48	52.2	40		0.420
	no	71	44.7	44	47.8	27		
Complications	retinopathy	14	8.8	9	9.8	5	7.5	0.779
	nephropathy	10	6.3	8	8.7	2	3.0	0.193
	neuropathy	16	10.1	8	8.7	8	11.9	0.596
	cardiovascular	18	11.3	11	12.0	7	10.4	0.806
	non	106	66.7	62	67.4	45	67.2	0.923
Therapy	exercise therapy	52	32.7	33	35.9	19	28.4	0.393
	diet therapy	71	44.7	47	51.1	24	35.8.	0.075
	insulin therapy	54	34.0	40	43.5	14	20.9	0.004*
Diabetes knowledge (the general knowledge subscale)	average (SD) [range]	9.8 (3.4) [0–16]		10.4 (2.4) [4–16]		8.9 (4.2) [0–16]		0.050
Number of medications	median [range]	5 [1–20]		5 [1–20]		5 [1–17]		0.577
*Number* of doses *per day*	median [range]	8 [0–50]		9 [1–50]		8 [0–28]		0.137
Patient’s perception	Factor 1. Feeling of inferiority	3.1 (2.3) [0–10]		3.0 (2.2) [0–8]		3.2 (2.5) [0–10]		0.745
	Factor 2. Living a tidy life	6.9 (2.1) [0–10]		7.1 (2.1) [0–10]		6.8 (2.1) [1–10]		0.195
	Factor 3. Feeling of restriction	5.2 (1.9) [0.2–9]		4.9 (1.9) [0.2–8.6]		5.5 (1.7) [1–9]		0.076
	Factor 4. Feeling miserable	5.3 (2.1) [0–10]		5.6 (2.1) [0–10]		4.9 (2.1) [0–9.67]		0.028
	Factor 5. Feeling of getting into trouble	3.9 (1.9) [0–9.67]		3.8 (1.7) [0–7.17]		3.9 (2.2) [0–9.67]		0.919
	Factor 6. Feeling of intemperance	6.2 (1.9) [0–10]		6.4 (1.7) [2.33–10]		6.1 (2.2) [0–9.33]		0.797
	Factor 7. Feeling of portentous	6.5 (2.0) [0.67–10]		6.8 (1.6) [2.33–10]		6.0 (2.3) [0.67–10]		0.013

^a^Mann–Whitney U-test or Fisher’s exact test was used to test the mean difference between individuals at hospital and community pharmacy; *Pearson’s* chi-square test was used to test the distribution difference of categories between individuals at hospital and community pharmacy

### Clustering medication adherence behavior

Table 2 shows the correlation between the total medication adherence score and patient characteristics. Significant relationships were found between medication adherence and BMI, family history of diabetes, diabetes knowledge, and two factors of patient’s perception (factor 2: living an orderly life and factor 6: feeling of overindulgence).

**Table 2:** The correlation between the total medication adherence score and patient characteristics

	Total medication adherence score	
	coefficient of Spearman’s correlation	*p*-value
Sex	0.018	0.821
Age (years)	0.098	0.217
BMI	−0.171	0.031*
Diabetes duration (years)	0.154	0.053
HbA1c (%)	− 0.041	0.658
Family history of diabetes	0.244	0.002*
Complications		
retinopathy	0.119	0.134
nephropathy	0.008	0.919
neuropathy	0.066	0.41
cardiovascular	0.136	0.088
non	−0.101	0.206
Therapy		
exercise therapy	0.068	0.397
diet therapy	0.112	0.159
insulin therapy	−0.029	0.72
Diabetes knowledge	0.169	0.033*
Number of medications	0.114	0.185
Number of doses per day	0.154	0.073
Patients’ perception		
Factor 1. Feeling of inferiority	−0.119	0.134
Factor 2. Living an orderly life	0.253	0.001**
Factor 3. Feeling of restriction	0.059	0.461
Factor 4. Feeling miserable	0.039	0.63
Factor 5. Feeling of getting into trouble	0.064	0.428
Factor 6. Feeling of overindulgence	0.205	0.009**
Factor 7. Feeling of importance	0.035	0.665

*significant at 0.05 level, **significant at 0.01 level

We investigated the profile of medication adherence in T2DM patients according to the medication adherence behavior. Considering that medication adherence has four subscale factors, the score for each subscale factor showed a characteristic for each patient with the same degree of adherence. Therefore, patients were classified into clusters by using a subordinate scale factor of medication adherence. We used PCA to reduce the number of medication adherence variables to a smaller number of independent dimensions. The varimax rotation was used to simplify the PC extracted, and the medication adherence between diabetic patients after the projection of variables and that of diabetic patients represented by components 1 and 2, respectively, were summarized**.** The corresponding eigenvalues give the information percentages explained by these factors, namely, 52.5 and 23.8% for diabetic patients after the projection of variables and diabetic patients represented by components 1 and 2, respectively) (Table 3). Table 3 also shows three factors of adherence: subscale factor 1: collaboration with healthcare providers; subscale factor 2: motivation for collecting and using medication-related information; subscale factor 3: agreement to take medication and its fit with their lifestyle, which contribute strongly to component 1; and, by contrast, subscale factor 4 (the current state of medicine use) provided a large contribution to component 2. Therefore, in this result, components 1 and 2 were respectively named as “accessibility to medical treatment” and “status of taking medicines” for convenience.

**Table 3:** Component loadings for medical adherence in varimax rotation principal components

	Component 1	Component 2
Subscale factor 1: collaboration with healthcare providers	0.77	−0.434
Subscale factor 2: motivation of collecting and utilizing medication-related information and utilization of information regarding medication	0.834	−0.34
Subscale factor 3: agreement with taking medication and its fit with their lifestyle	0.73	0.311
Subscale factor 4: the current state of medication use	0.526	0.742
% cumulative variance	52.5	23.8

Positive signs indicate that higher values of the variable are influential in the component, whereas negative signs indicate the influence of lower values

**Fig. 1:**
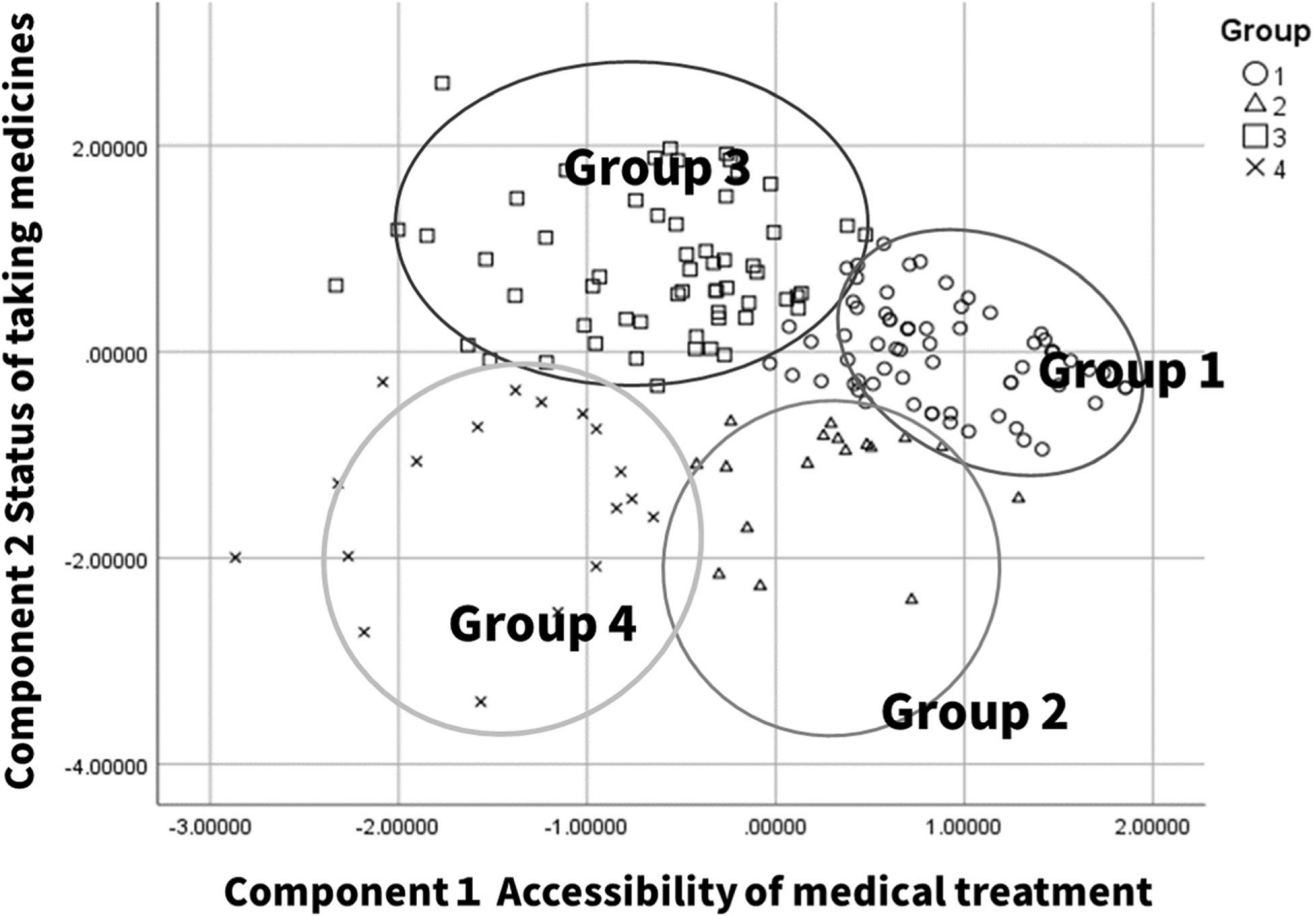
Projection of variables on the first plan given by Principal Component Analysis (PCA)

The scores of these two principal components were used for the cluster analysis (CA) by dividing the patient cohort into four groups. Table 4 shows the CA comparison of the four groups of variables. The first group (group 1) was characterized by high accessibility to medical treatment, the second group (group 2) demonstrated medium accessibility to medical treatment, the third group (group 3) had a high level of adherence for taking medicines, and the fourth group (group 4) showed low medication adherence. Figure 1 shows the scatter plot of all the patients. The X-axis (component 1) is mainly determined by variable prevalence and accessibility to medical treatment, whereas the Y-axis (component 2) is mainly determined by the level of medicine adherence (the variable adherence). Table 5 shows the patient characteristics for each group. The age, BMI, family history of diabetes, and the two factors of a patient’s perception (factor 2: living an orderly life and factor 6: feeling of overindulgence) of this cohort were significantly different in the four groups. The characteristics of the groups with good adherence (group1 and 3) were older and lower BMI than that of the poor adherence group. Moreover, patients in the good adherence groups have strong tendencies to live an orderly life and overindulgence about diabetes.

**Table 4:** Defining characteristics of the clusters

Medication adherence	Group 1 (*n* = 58)	Group 2 (*n* = 17)	Group 3 (*n* = 66)	Group 4 (*n* = 18)
Component1 Accessibility to medical treatment	0.87086	0.26542	−0.61106	−1.47487
Component 2 Status of taking medicines	−0.01329	−1.22898	0.82345	−1.44393

**Table 5:** Patient characteristics for each group

		Group1		Group2		Group3		Group4		*p*-value^a^
		*n*	%	*n*	%	*n*	%	*n*	%	
Gender	male	45	68.2	13	76.5	40	69.0	12	66.7	0.917
	female	21	31.8	4	23.5	18	31.0	6	33.3	
Age (year)	median	68	(10.3)	65	(11.6)	69	(10.7)	60	(13.6)	0.011*
	[range]	[34–88]		[32–76]		[42–88]		[35–83]		
BMI	median [range]	23.4 [15.2–34.8]		23.5 [15.9–29.6]		24.7 [18.6–34.0]		26.2 [17.7–32.7]		0.014*
Diabetes duration (year)	median [range]	15 [0–53]		10 [1–25]		11 [0–55]		10 [1–25]		0.270
HbA1c (%)	median [range]	7.0 [4.4–9.2]		7.4 [5.6–10.0]		6.9 [5.9–10.6]		7.4 [5.8–9.9]		0.111
Diabetes history of relatives of Relatives	yes	45	68.2	10	58.8	24	41.4	9	50.0	0.026*
	no	21	31.8	7	41.2	34	58.6	9	50.0	
Complications	Diabetic retinopathy	8	12.1	1	5.9	5	8.6	0	0.0	0.419
	Nephropathy	4	6.1	0	0.0	6	10.3	0	0.0	0.264
	Neuropathy	8	12.1	3	17.6	5	8.6	0	0.0	0.316
	Heart disease	12	18.2	1	5.9	4	6.9	1	5.6	0.150
	none	41	62.1	12	70.6	37	63.8	17	88.9	0.176
Therapy	Exercise therapy	22	33.3	5	29.4	18	31.0	7	38.9	0.923
	Dietary Therapy	29	43.9	10	58.8	25	43.1	7	38.9	0.638
	Insulin therapy	20	30.3	8	47.1	18	31.0	8	44.4	0.424
Number of medications	median [range]	5 [5.6–10.0]		5 [1–13]		5 [1–15]		6 [2–11]		0.849
*Number* of doses *per day*	median [range]	9 [1–28]		7 [1–23]		8 [0–50]		8 [2–20]		0.543
Diabetes knowledge	average (SD) [range]	10.5 (2.9) [0–16]		10 (2.1) [5–13]		9.0 (4.0) [0–15]		9.3 (3.3) [0–14]		0.321
Patient’s perception	Factor 1. Feeling of inferiority	2.9 (2.3) [0–8]		2.9 (2.0) [0–6.6]		3.1 (2.4) [0–10]		3.8 (2.3) [0–8]		0.515
	Factor 2. Living a tidy life	7.5 (1.8) [3–10]		6.6 (1.2) [5–9]		6.8 (2.3) [0–10]		5.6 (2.4) [1–9.33]		0.012*
	Factor 3. Feeling of restriction	5.3 (1.9) [0.8–9]		5.9 (1.7) [1–7.8]		5.0 (1.9) [0.2–8]		4.8 (1.9) [1–7.4]		0.209
	Factor 4. Feeling miserable	5.3 (2.1) [0–9.33]		5.4 (1.8) [2.33–9.33]		5.4 (2.3) [0–10]		5.1 (2.0) [1.67–9]		0.980
	Factor 5. Feeling of getting into trouble	4.0 (2.2) [0–9.33]		4.2 (1.7) [0.83–7]		3.9 (1.7) [0.5–9.67]		3.1 (1.6) [0–6.33]		0.282
	Factor 6. Feeling of overindulgence	6.7 (1.9) [0–10]		6.2 (1.7) [4–10]		6.0 (1.9) [0.67–9.33]		5.4 (1.9) [2.33–9]		0.044*
	Factor 7. Feeling of portentous	6.4 (1.8) [2–10]		7.3 (1.4) [4.33–10]		6.5 (2.3) [0.67–10]		5.9 (1.9) [2–9]		0.181

^a^Kruskal–Wallis test was used to test the mean difference among four groups. *Fisher’s* exact test wa*s* used to test the distribution difference of categories between individuals at the hospital and the community pharmacy. BMI, body mass index. *significant at 0.05 level

### Association between medication adherence and demographic/clinical characteristics, illness perceptions about diabetes, and diabetes-specific knowledge in patients

Multiple regression analysis was performed to analyze predictors of medication adherence. BMI, family history of diabetes, one factor of patient’s perception (factor 2: living an orderly life), and diabetes knowledge were found to be significant predictors of medication adherence of patients with T2DM (Table 6).

**Table 6:** Multivariable regression analysis for medication adherence

Variables	B	SE	β
Patients’ perception factor 2: Living an orderly life	0.970	0.307	0.239**
Family history of diabetes	3.392	1.250	−0.198**
Diabetes knowledge	0.504	0.192	0.199**
BMI	−0.391	0.170	−0.17*

*BMI* body mass index, B, partial regression coefficient for the constant in the null model. SE, the standard error around the coefficient for the constant. β, standard partial regression coefficient. *Significant at 0.05 level. **Significant at 0.01 level. The coefficient of determination (R2) in this regression equation was 0.195

The explanatory variables of interest were BMI, family history of diabetes, patient’s perception, and diabetes knowledge. Table 7 presents results of the multinomial logistic regression analysis of factors associated with adherence to medications by using group 1 (high level of adherence to medications) as a reference. There was a significant correlation between patients’ perception of disease/diabetes knowledge and medication adherence. A low level of adherence to medications (group 4) was associated with patient’s perception (odds ratio (OR) = 0.697; 95% confidence interval (CI): 0.523–0.930). A medium level of adherence to medications (group 3) was associated with high BMI (OR = 1.159; 95% CI: 1.034–1.300) and poor diabetes knowledge (OR = 0.844; 95% CI: 0.741–0.961).

**Table 7:** Multinomial logistic regression results in medication adherence groups

Variables	B	Odds ratio	95% confidence interval	
			lower	upper
Group 2				
Age	−0.055*	0.946	0.899	0.996
BMI	−0.078	0.925	0.787	1.088
Family history of diabetes	−0.350	1.419	0.447	4.506
Patients’ perception factor 2: living an orderly life	−0.257	0.773	0.572	1.045
Diabetes knowledge	−0.049	0.952	0.789	1.150
Group 3				
Age	0.000	1.000	0.963	1.040
BMI	0.148*	1.159	1.034	1.300
Family history of diabetes	−1.250**	3.490	1.580	7.709
Patients’ perception factor 2: living an orderly life	−0.121	0.886	0.722	1.087
Diabetes knowledge	−0.169*	0.844	0.741	0.961
Group 4				
Age	−0.072**	0.931	0.882	0.982
BMI	0.122	1.130	0.961	1.328
Family history of diabetes	−0.618	1.855	0.552	6.239
Patients’ perception factor 2: living an orderly life	−0.361*	0.697	0.523	0.930
Diabetes knowledge	−0.180	0.835	0.693	1.006

BMI, body mass index; B. Partial regression coefficient; The coefficient of determination (R^2^) in this regression equation was 0.273. *significant at 0.05 level, **significant at 0.01 level

## Discussion

We found that our study T2DM cohort was to categorize to four distinct subphenotypes according to a behavioral subphenotypes of medication adherence in T2DM patients using a validated medication adherence scale and moreover the good adherence group was significantly associated with the patient’s perception of “living an orderly life.”

Diabetic patients adherence to their medication schedules more closely if they believe in medication efficacy and perceive their illness as manageable [29–34]. Patients with hypertension strongly believe that hypertension can be controlled by medical treatment [35, 36] or by managing their diets and lifestyle [37]; therefore, they strongly adhere to antihypertensive medications. Satisfaction, convenience, and effectiveness were associated with a good medication adherence in patients with dyslipidemia [38].

Measuring patient perception by Kamatani’s method showed that diabetic patients who lived an orderly life had good medication adherence. Better adherence might be related to beliefs in medication efficacy and illness perceptions. Patients’ perceptions change their viewpoint regarding life with T2DM, i.e., whether life goes forward depends on the disease or on their own initiative to live life responsibly. We hypothesized that positive recognition of diabetes and/or understanding of diabetes could be positively associated with the good medication adherence of T2DM patients. Therefore, we suggested devising strategies to promote illness perception that will help patients develop a customized diabetes health plan.

As another predictor of medical adherence in T2DM patients, BMI, diabetes knowledge, and family history of diabetes were identified. Tominaga et al. reported that older age was significantly associated with better medication adherence [39]. A high BMI (obesity) and a family history of diabetes are significantly and positively associated with the risk of T2DM progression [40–42]. A previous report showed that a family history of DM is associated with lower physical activity and noncompliance with dietary advice [43–45]. Diabetic patients’ knowledge of their disease is one of the important determinants of self-management practices. This relationship is very important in diabetes intervention for medical providers and patients because knowledge of diabetes and self-management practice are significantly related to glycemic control [46, 47].

There is a correlation between disease perception and health outcomes because self-management is complex. It involves complicated decision making that depends on the patients’ perception of their illness in terms of whether it is controllable, understandable, curable, or serious [48–50]. We hypothesize that a poor adherence group can change their adherence to diabetes treatment by developing the perception of “living an orderly life.”

This study has several limitations. The responses from the questionnaires were based on self-declaration; therefore, they are prone to potential errors and misunderstanding of the questions. In particular, the results of medication adherence may be subjected to recall bias and social desirability bias, especially when it comes to sensitive questions such as medication adherence. A cross-sectional design with convenience sampling was adopted in this study. There is a difference in the baseline characteristics of patients in the hospital vs. community settings, such as age and gender, considering that patients who have been visiting a pharmacy for a long time might readily agree to participate in this study. This could be a limitation as selection bias might have occurred for participants from the pharmacy.

## Conclusions

We found that medication adherence in patients with T2DM is presumed by BMI, diabetes knowledge, family history of diabetes, and the diabetic patient’s perception of “living an orderly life.” Patients who have a perception of “living an orderly life” have good medication adherence. It may be beneficial to tailor health risk communications targeting T2DM to match the recipients’ personality characteristics instead of using the “one-size-fits-all” approach. Future prospective studies are required to confirm the therapeutic effects of behavioral interventions for the perception of diabetes.

## Additional files

Additional file 1: **Table S1.** Medication adherence questionnaire. (DOCX 16 kb)

Additional file 2: **Table S2.** Patients’ perceptions of diabetes questionnaire. (DOCX 16 kb)

Additional file 3: **Table S3.** Revised Michigan Diabetes Knowledge Scale (DKT) - True/False Version. (DOCX 15 kb)

## Abbreviations

BMI: Body mass index
DKT: Michigan Diabetes Knowledge Scale
PCA: Principal-component analysis
T2DM: Type 2 diabetes
