# Supplementary material for: The relationship between patients’ perception of type 2 diabetes and medication adherence: a cross-sectional study in Japan
Source: J Pharm Health Care Sci. 2019 Jan 22;5:2. doi: 10.1186/s40780-019-0132-8 (PMC6341584; doi:10.1186/s40780-019-0132-8)
Supplement: Supplementary file 1 — Table S1. Medication adherence questionnaire. (DOCX 16 kb) [file 40780_2019_132_MOESM1_ESM.docx]

**Table S1. Medication adherence questionnaire**

The following is a list of questions regarding your medication adherence. Please read each question and rank your response on the five-point scale directly below each statement, with “1” indicating that you strongly disagree with the statement and “5” indicating that you strongly agree with the statement.

Additional space is also provided for your comments on each statement.

I. Collaboration with healthcare providers (doctor, pharmacist, nurse, etc.)

1. I can ask questions about the medication without hesitation to medical staff.
2. I can share my expectations regarding therapy and treatment goals with medical staff.
3. I can share treatment progress regarding medication drug therapy with medical staff.

II. Motivation of collecting and utilizing medicine-related information and utilization of information regarding medication

1. I can ask medical staff to explain what I do not understand about my medication.
2. I inform medical staff if my medicine does not seem to be working or if it is causing problems (side effects, allergy symptoms, etc.).
3. I know the name of the drug and understand why it has been prescribed for me, and I know about the importance of taking all your medicines.
4. I can improve my condition on my own by taking my medication.
5. I am searching for information about my medicines to avoid possible problems.

III. Agreement with taking medication and how it fits with your lifestyle

1. I am convinced of the importance of my prescription medication.
2. I take medication as a part of my lifestyle, such as eating meals and brushing my teeth.
3. I have no difficulty accepting support from my family and friends about taking all my medications, as advised by my physician.

IV. About the current state of medication use

1. I take the medications according to the number of times per day stated in the instructions.
2. I take my medications every day automatically at a fixed time or on fixed intervals.
3. I do not stop taking my medication just because I want to.
