# Supplementary material for: The relationship between patients’ perception of type 2 diabetes and medication adherence: a cross-sectional study in Japan
Source: J Pharm Health Care Sci. 2019 Jan 22;5:2. doi: 10.1186/s40780-019-0132-8 (PMC6341584; doi:10.1186/s40780-019-0132-8)
Supplement: Supplementary file 2 — Table S2. Patients’ perceptions of diabetes questionnaire. (DOCX 16 kb) [file 40780_2019_132_MOESM2_ESM.docx]

**Table S2. Patients’ perceptions of diabetes questionnaire**

The following are 28 statements about patients’ perceptions of diabetes. Please read each statement and rank your response on the 11-point scale directly below each statement, with “0” indicating that you strongly disagree with the statement and “10” indicating that you strongly agree with the statement.

1. The complications of diabetes seem horrible.
2. Fat people get diabetes.
3. Sweets and greasy foods may cause diabetes.
4. A specific lifestyle may cause someone to get diabetes.
5. The body will gradually lose mobility because of the complications of diabetes.
6. Diabetes causes me to feel bad.
7. Diabetes does not cause harm.
8. Diabetes is a terrible disease.
9. Diabetes is not an illness for which one dies alone.
10. All I can do is not make it worse.
11. I cannot eat anything the same way as another person.
12. Diabetes causes sorrow.
13. Diabetes is determined [by](http://english.cheerup.jp/dict/search?name=by) our genetics.
14. I am not motivated to treat my diabetes properly.
15. Insulin-requiring states for treating diabetes are severe.
16. I feel that I will waste all of my life when I think about having/living with diabetes.
17. I feel embarrassed about my diabetes.
18. Diabetes may cause serious trouble for me.
19. I feel incompetent as a man with diabetes.
20. Another person [has](http://english.cheerup.jp/dict/search?name=have) [no](http://english.cheerup.jp/dict/search?name=no) [reason](http://english.cheerup.jp/dict/search?name=reason) to complain about my diabetes.
21. I am labeled a failure.
22. I feel alone with diabetes.
23. I am grateful for my situation having/living with diabetes.
24. I feel that having/living with diabetes is my destiny.
25. I cannot understand diabetes.
26. Diabetes is a bad friend.
27. Daily routines are regulated by having/living with diabetes.
28. I feel quite safe when I manage my diabetes well.
29. I am constantly concerned about food and eating.
