# Supplementary material for: The relationship between patients’ perception of type 2 diabetes and medication adherence: a cross-sectional study in Japan
Source: J Pharm Health Care Sci. 2019 Jan 22;5:2. doi: 10.1186/s40780-019-0132-8 (PMC6341584; doi:10.1186/s40780-019-0132-8)
Supplement: Supplementary file 3 — Table S3. Revised Michigan Diabetes Knowledge Scale (DKT) - True/False Version. (DOCX 15 kb) [file 40780_2019_132_MOESM3_ESM.docx]

**Table S3.** **Revised Michigan Diabetes Knowledge Scale (DKT) - True/False Version.**

Here are 20 statements about diabetes, some are true statements and some are false. Please read each statement and then indicate whether you think it is true or false by putting a circle round either TRUE or FALSE. If you do not know the answer please put a circle around DON’T KNOW.

1. The diabetes diet is a healthy diet for most people

2. Glycosylated haemoglobin (HbA1c) is a test that measures your average blood glucose level in the past week.

3. A pound of chicken has more carbohydrate in it than a pound of potatoes.

4. Orange juice has more fat in it than low fat milk.

5. Urine testing and blood testing are both equally as good for testing the level of blood glucose.

6. Unsweetened fruit juice raises blood glucose levels.

7. A can of diet soft drink can be used for treating low blood glucose levels.

8. Using olive oil in cooking can help lower the cholesterol in your blood.

9. Exercising regularly can help reduce high blood pressure.

10. For a person in good control, exercising has no effect on blood sugar levels.

11. Infection is likely to cause an increase in blood sugar levels.

12. Wearing shoes a size bigger than usual helps prevent foot ulcers.

13. Eating foods lower in fat decreases your risk for heart disease.

14. Numbness and tingling may be symptoms of nerve disease.

15. Lung problems are usually associated with having diabetes.

16. When you are sick with the flu you should test for glucose more often.

SKIP TO QUESTION 19 IF YOU DON’T TAKE INSULIN

17. High blood glucose levels may be caused by too much insulin.

18. If you take your morning insulin but skip breakfast your blood glucose level will usually decrease.

19. Having regular check-ups with your doctor can help spot the early signs of diabetes complications.

20. Attending your diabetes appointments will stop you getting diabetes complications.
